# Understanding gender inequity in brain health outcomes: missed stroke as a case study for intersectionality

**DOI:** 10.3389/fgwh.2024.1350294

**Published:** 2024-02-12

**Authors:** Suze G. Berkhout, Syeda Hashmi, Aleksandra Pikula

**Affiliations:** ^1^ University Health Network, Toronto, ON, Canada; ^2^ Department of Psychiatry, University of Toronto, Toronto, ON, Canada; ^3^ Jay and Sari Sonshine Centre for Stroke Prevention and Cerebrovascular Brain Health, Toronto, ON, Canada; ^4^ Division of Neurology, University of Toronto, Toronto, ON, Canada

**Keywords:** stroke, gender, intersectionality, misdiagnosis, brain health gap, implicit bias

## Abstract

Recent attention into sex and gender-based inequities surrounding outcomes for brain health disorders has generated momentum toward addressing what has been called the “brain health gap.” Importantly though, “women” are not uniform demographic group. In this perspective piece, we discuss misdiagnosis in stroke as an aspect of access and quality of care within brain health. Drawing on narrative data from a mixed methods study of young stroke survivors we suggest that while missed stroke isn't only an issue of gender, if we are going to understand gender-based gaps in access and navigation through stroke care, we have to understand how intersections of gender with age, ethnoracial identity, nationality, language, (dis)ability, and other aspects of social identity come together to create affordances as well as biases that contribute to stroke outcomes.

## Introduction

The one thing I keep going back to is the night of the stroke, in the first hospital, the nurses clearly didn’t recognize the symptoms. Or, they didn’t recognize the symptoms in a younger female. That, to me, is intensely problematic for very obvious reasons. The whole experience was so bad that I was actually encouraged by one of my neurologists in [that city] to launch a complaint.

(Ms. G, Y-Stroke Needs Participant)

Recent attention into sex and gender-based inequities surrounding outcomes for brain health^1^ disorders has generated momentum toward addressing what has been called the “brain health gap”. From translational research to health policy and structural change, disparities in understanding the prevalence, detection, and treatment of many forms of brain-related illness that disproportionately impact women have received increased attention (1). Mechanisms for changing these disparities are increasingly called in (2–4). Importantly though, “women” are not uniform demographic group. Various scholarly fields—notably within Black Feminist and critical race scholarship, and more recently what has been termed “crip of colour” critiques from within disability studies^2^—have highlighted the ways in which the intersections *and* interactions of racism, patriarchy, ableism and heteronormativity (amongst other dimensions of social power) are crucial for understanding and addressing health-related inequity (5, 6).

Depending on how they are positioned by virtue of their social identities, some individuals struggle with ableism and expectations of normative embodiment. Navigating medical care can often mean navigating medicalization and ableist assumptions that pathologize diversity in lived experience (7). For others, though, just getting a foot in the door—accessing care *at all*—becomes the issue (6, 8). When lived experiences are shaped by intersections of social power and minoritized group memberships, health care may be withheld, withdrawn, or offered on terms that are low quality or compromise one's values and integrity (9). Understanding how these different dynamics contribute to gender inequity in brain health—the brain health gap—requires an approach oriented within intersectionality. Inequities in relation to access and quality of care within brain health exist not only along lines of gender: we need to consider gender as imbricated with race, ethnicity, sexuality, economic background, (dis)ability, age, geography, and religion, as well as other sources of discrimination and subordination.

Intersectionality is both a theoretical framework and a research praxis that understands inequity in relation to the dynamics of difference and sameness, which impact people by virtue of their membership in social groups; it is an approach explicitly oriented toward social justice (5, 10, 11). An intersectional analysis offers a way of thinking about how social axes of power impact individuals vis a vis their multiple social identities (12, 13). Within the healthcare system, these overlapping (and often mutually constitutive) systems of disadvantage and/or privilege shape experiences of care. In the context of stroke diagnosis and post-stroke care, these overlapping identities can result in clear markers of advantage for some, while manifesting disparities in outcome due to bias and structural forms of disadvantage for others (14).

Drawing on our own mixed-methods research exploring the care needs of young stroke survivors (*Y-Stoke Needs* study; funded by the Canadian Institutes of Health Research and approved by University Health Network research ethics board), we turn to the narrative of Ms. G: a woman in her early forties with a missed diagnosis of stroke. Ms. G's symptoms were ignored or challenged by medical professionals because she did not fit the template of who a stroke patient was and because some aspects of her identity delimited the extent to which her testimony about her symptoms was taken as credible. And yet, Ms. G's social position was also marked, at least in some ways, by privilege. Her story provides a foundation for helping us to think about the complexities of the brain health gap in stroke.

More women than men suffer cerebrovascular accidents (CVAs) and women's outcomes are often worse—they are treated less aggressively for primary and secondary prevention; they are more likely to have lower quality of life post-stroke as well as higher prevalence of post stroke psychiatric comorbidities (15–17). But as we argue, we need to think intersectionally to understand the ways in which nuances, contexts, and multi-level factors come together to shape these broad-based inequity findings. Missed stroke isn't *only* an issue of gender—if we are going to understand gender-based gaps in access and navigation through stroke care, we have to understand how intersections of gender with age, ethnoracial identity, nationality, language, (dis)ability, and other aspects of social identity come together to create affordances as well as biases that contribute to stroke outcomes.

## The case

When our team met her, Ms. G was a single woman in her early forties, a well-educated scientist working in a provincial public service position. She had been away on a work trip when, three days into the trip, she experienced a sudden onset of gastrointestinal symptoms late one night. As she described to us, she realized something was “very wrong” when what seemed like typical nausea and vomiting was suddenly accompanied by a rapid and progressive loss of sensation and motor control in her right leg.

The day of the stroke, I had felt off. Off, in the sense that I felt like maybe I was getting sick, that something was coming… I felt so rundown that I declined going out with [my colleagues] and I thought I would just have a quiet night. I continued to not feel terribly well throughout the course of the night and I ended up going to bed a little bit early, around 10:00. Then, I woke up at 11:00, with an intense need to vomit… Then, around 2:00, I started to realize that something was very, very wrong, because I had lost feeling in my right leg. It started as a tingle and then it progressed into full paralysis of my right leg… I was starting to suspect I was having a stroke, I ended up calling 911. I told the ambulance attendant that I couldn't move my right leg and we went to the hospital. That's where the story gets really bad. The nurses that I was assigned did not believe me when I told them that I had lost feeling in my right leg. They thought that I was lying to them and were refusing care to me. (Ms. G)

Our team met Ms. G less than one year from her stroke and she was doing well, all things considered. She had access to rehabilitation, she was supported with workplace accommodations, she was managing mood and cognitive symptoms with multidisciplinary supports.

The *Y-Stroke Needs* study aims to understand the challenges facing young stroke survivors, from the onset of symptoms, through acute care and the rehabilitation process, to long-term survivorship (UHN REB #17-6092; all study participants provided written informed consent). Ms. G participated in the qualitative arm of the project, sharing with us her narrative of the experiences she had as she moved through the post-stroke pathways within the Canadian healthcare system. Even for as much as her outcomes were positive, overall, the experience of accessing stroke care had been marked by distress.

At one point in time, I was so desperate for water, and I knew that the water fountain was directly next to my bay, I decided to try and walk there. Forgetting, of course, that my right leg was paralyzed. As soon as I tried to stand up, I hit the ground. That's when one of the nurses told me outright that I was lying, that she had seen me move my leg and since I had put myself onto the ground, I could get myself back up. So, I tried to do that, and I ended up falling backwards and dislocating my thumb. Which she then accused me of lying about. She told me my thumb was just double-jointed and that it could move back. I was in the ER of [the general hospital] from 4:00 in the morning until noon. At noon, I was transferred out to the designated stroke hospital in [that city's] system. (Ms. G)

Despite symptoms typical of public education campaigns and infographics on stroke (e.g. sudden onset of unilateral weakness), her symptoms were minimized, unrecognized, and mischaracterized, putting Ms. G well beyond the optimal window for acute stroke identification and initial management. The challenges and issues prompted by her experience are not simply related to a misdiagnosis through the inevitabilities of human error or the subtleties and evolution of clinical signs and symptoms.

Ms. G had articulated and displayed physical signs and symptoms of stroke: these were interpreted by healthcare providers through a lens that could not make sense of these *as* stroke symptoms, because of her intersecting social identities. A younger, single, white woman, her exam findings were read as anxiety at best and manipulation at worst—a throwback to categorizations of hysteria that continue to impact the uptake of women's embodied experiences in the healthcare system (18). The enduring legacy of hysteria as a label for women's health concerns highlights the persistent gender biases within the healthcare system. Relegating symptoms to historic stereotypes risks overlooking legitimate health issues, perpetuating a cycle of disbelief that can impact the quality and timeliness of care. Acknowledging this historical context is crucial for dismantling stereotypes and ensuring a more equitable and compassionate approach to women's health, rooted in evidence-based medicine and a genuine understanding of diverse experiences.

## Epidemiology of misdiagnosis in stroke: a case for intersectionality

I literally told the EMT who picked me up, and this is a quote, “it's like my brain is sending signals that my foot isn’t responding to”. And I don't know how that information didn't get to the nurses who were responsible for my care. It seems to me that was pretty self-explanatory what it meant. But I think the one thing, people really need to understand the signs of stroke in younger women. My nurses, I know I told you this, but they not only didn't believe me—they accused me of lying about my symptoms. And that, to me, is unconscionable. (Ms. G)

Like Ms. G, women who present with stroke are more likely to have their symptoms go unrecognized; variations exist in the timeframe at which women receive standardized and evidence-based care and the type of care offered compared to men, including being less likely to be seen by a stroke specialist or receive diagnostic testing (19, 20). The interplay between gender and adherence to guidelines is also rapidly evolving: in 2018, two-thirds of heart and stroke clinical research was reported to be based on symptoms in men; 28% of women received ECG within 10-min period in contrast to 38% of men; clot-dissolving therapy (within the recommended 30-min period) was offered to 32% of women in contrast to 59% of men (21). More recently though, we see significant geographic differences and a multiplicity of factors underlying in gender inequity in the detection of stroke (22) as well as increasing sex-based parity within time trends in endovascular therapy (23). Gender bias-based “knowledge gaps” (24) are variable in how they translate to clinical disparities in prevention, diagnosis, post-stroke care and secondary outcome within the dynamic relationships between age, gender, ethnoracial identity, language and nationality (25, 26).

Social ecological models of disparities relating to access to stroke care and functional outcomes from stroke are particularly demonstrative of the ways that gender, ethnoracial minoritization and class/socioeconomic status are mutually constitutive of increased barriers and worsened outcomes (27). Epidemiological data relating to inequity and health disparities in stroke are well-documented, but understanding underlying causes has been more lacking, often due to the complex and multi-level nature of the phenomena: intra- and interpersonal factors including implicit bias and stereotype threat; institutional and organizational factors such as the number of care transitions that take place in stroke pathways; multidirectional neighborhood and community factors that influence predisposing factors in addition to accessibility of care, referral pathways, and functional supports; and larger policies and practices that can embed structural forms of racism amongst other discriminatory practices in health settings (27).

I had to wait until the ER doctor came to see me, which was, to the best of my recollection, was about 9:00 a.m. The ER doctor did a quick reactivity test on my foot, realized that there was absolutely no reaction and immediately sent me for a CT scan. That's when they found the two bleeds. He also attempted to push my thumb back into place, without anaesthetic, which caused me to scream very loudly. It was quite dislocated. So, I certainly hadn't made up that injury. To this day, I still don't have full functionality back in my thumb. (Ms. G)

While the interpersonal factors that Ms. G cogently described are notable within this particular case, we also have to consider how larger systems and structures facilitate (or alternatively can correct) implicit biases. It is inadequate to attend only to individual-level factors in understanding what hinders timely and accurate diagnosis and treatment. Her younger age and a combination of typical as well as “atypical” symptoms decreased attention to stroke as a possibility. Young people have higher occurrence of less typical stroke symptoms and greater heterogeneity in stroke etiology; this is especially true for women (28, 29). But rather than consider that age and gender might lead to the presence of less typical stroke symptoms, in this case the intersection (particularly with gender) contributed to the characterization that Ms. G was not straightforward or was mistaken in her depiction of these symptoms. This reflects how “typical” symptoms have been determined based on older (usually male) bodied experiences, which get set as the unmarked norm; it also reflects what Maya Dusenbery has termed the “trust gap” that operates in healthcare settings (24). The “trust gap” refers to a tendency to treat particular group members as less credible in their testimony or interpretation of their own experiences, contributing to a dismissal or minimization of symptoms, under-treatment, and misdiagnosis.

The “trust gap” is lockstep with knowledge gaps and can be understood as contributing to what has been termed *epistemic injustice*—a form of injustice in which particular group members are regarded as less credible or knowledgeable about their own situation due to their social position qua group member (30). Epistemic injustice exacerbates negative psychosocial impacts of medical experiences, affecting a person's sense of self, their ability to trust their own judgments, and their recovery process (31), while further contributing to asymmetries of knowledge/power within medical contexts (32).

Once I was transferred to the designated stroke hospital, my care improved significantly… I was [also] in rehab for about a month… My stay [in rehab] was great, everyone there was fantastic… At the point when I was discharged, I was walking with a cane. Then, about a week after I was discharged, I was able to stop using the cane completely. At this point in time, my leg is completely recovered. I still have a little bit of the frozen arm thing happening. I can almost get my arm up, but not quite yet. But, it's improved quite a lot. The only other major effect that I’m feeling is a bit of short-term memory loss…

I'd say, there needs to be more understanding, awareness and recognition of what stroke looks like in younger people. They let me sit in the ER department for five hours, without doing any sort of neurological assessment. Had I had a clot-based stroke, my outcome would be very different right now. I'm extremely lucky that it was a hemorrhagic stroke. (Ms. G)

Importantly, Ms. G *also* occupied social positions of privilege—white, fluent in the language, higher socioeconomic group, employed—and so she was also able to advocate for herself once out of the emergency area. Her experience of being misdiagnosed and experiencing the trust gap about her symptoms was certainly distressing but was not repeated in numerous other health contexts she interfaced with. It did not stop her from accessing further rehabilitation services. Care transitions are an identified area where stroke survivors from historically disadvantaged groups are likely to face challenges (33). Ms. G's social identities contributed to misdiagnosis, but they became assets as she transitioned out of the initial healthcare setting, reflecting the dynamic nature of intersectionality.

## What does intersectional brain health look like?

**Figure 1:**
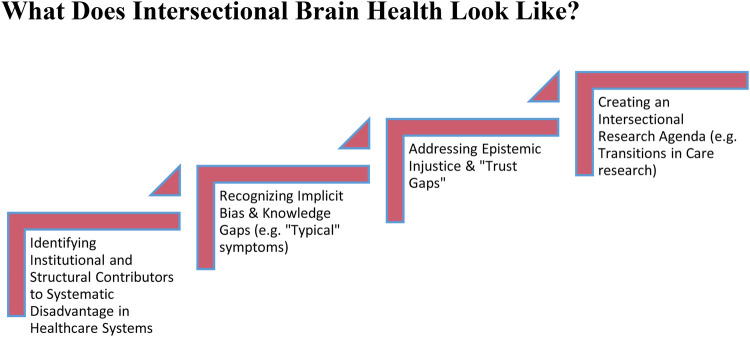
Agenda for intersectional practices in stroke care.

Our discussion illuminates a number of different issues, if the “brain health gap” relating to stroke is going to be addressed (Figure 1). First, a more nuanced understanding of challenges entering stroke pathways is needed, including the ways that misdiagnosis in stroke among minoritized groups impacts downstream care. The field needs to move beyond incidence rates for discrete demographic groups and understand differences *within* groups are being mediated by aspects of identity that are impacted by structural disadvantage (26, 34). The tendency to misdiagnose a stroke does not necessarily stem from a lack of technological advancements or incompetency among healthcare professionals. Rather, it arises from a range of institutional and structural factors that may include implicit biases, knowledge gaps surrounding who is impacted by less typical stroke symptoms and in what ways, *why* certain symptoms are considered less typical, and the systemic propensity to overlook or downplay symptoms in marginalized groups. The ongoing disparities amongst minoritized groups in stroke reflect an urgent need for an awareness and understanding of how intersectionality impacts clinical acumen, differential diagnosis, and access to high quality care.

Second, understanding and addressing the broader impact of stroke on women, younger adults, and people racialized as minorities extend beyond the initial misdiagnosis challenge that intersectional frameworks can inform. Stroke survivors are confronted with a complex array of health outcomes impacting their physical well-being, mental health and cognition (14), which increase a person's interfacing with medical care and the need for care transitions. Barriers and facilitators of recovery need an intersectional framework for research and care delivery given that ethnoracial minoritization, gender, age, language, (dis)ability, geography and nationality are all implicated as meaningful and context dependent. To that end, it is relevant that Ms. G was seen in the Canadian healthcare context—a (mostly) universal health system where acute care has substantive financial investment and where there is a degree of geopolitical stability. Understanding that the Canadian context is but one location, which will shift the valence of different social identities and their impact on stroke care is also crucial for ensuring that findings from one time and space are not erroneously generalized.

Finally, intersectionality calls us in to social change: research that is merely descriptive will not shift us toward a more inclusive model of healthcare that can reduce the incidence of misdiagnosis, improve treatment outcomes, and improve quality of life from a biopsychosocial perspective. Biases that contribute to misdiagnosis, lack of evidence-based intervention, and worsened longer-term outcomes are often more impactful for stroke patients who are minoritized along multiple social axes of power (14). Given that age-related biases are mutually constitutive with other forms of biases that influence stroke care (including gender bias), we need to ensure that attention is not merely paid to interpersonal processes and knowledge gaps, but rather that the larger underlying structural causes of these knowledge gaps are addressed (e.g., long-standing research practices that marginalize research into the health of minoritized groups as “special interest” rather than good science). When we fail to identify and name structural injustices *as* structural (for instance, by focusing on the individual encounter), epistemic marginalization takes place that furthers worse outcomes and the systems that contribute to inequity (35–37).

## Data Availability

The raw data supporting the conclusions of this article will be made available by the authors, without undue reservation.

## Footnotes

^1^We use the term “brain health disorders” and “brain health gap” in reference to a contemporary discourse around neurological experiences and medical care, but do so with the understanding that *health* is a nebulous and contested concept that often embeds normative assumptions about bodied and minded experiences that are themselves not neutral or based in matters of fact. See, for instance, *Against Health: How Health Became the New Morality*. Metzl and Kirkland, editors. NYU Press (2010).

^2^The concept of “crip” (invoked within the notion of “crip of colour critique”) makes reference to an orientation in critical disability studies that attends to insights from queer theory, exploring how gender/sexuality norms intersect with social pressures and norms relating to ability. Crip-of-colour scholarship further layers intersections of critical race theory within this orientation.
